# Exploring Pathways to Autism Spectrum Diagnosis in Adulthood

**DOI:** 10.1192/bjo.2024.140

**Published:** 2024-08-01

**Authors:** Isabelle Dufour, Sébastien Brodeur, Yohann Chiu, Émilie Dubé, Mélanie Couture

**Affiliations:** 1 Université de Sherbrooke, Sherbrooke, Canada; 2 Université Laval, Québec, Canada

## Abstract

**Aims:**

Autism spectrum is a neurodevelopmental condition usually diagnosed in early childhood. The broadened diagnostic criteria of the DSM–5 (2013) have led to an increasing number of autism spectrum diagnoses of individuals requiring lower levels of support. Barriers to diagnosis, especially in adults, include the complexity of differential diagnosis with co-occurring psychiatric disorders. This study explored the various pathways of psychiatric diagnosis preceding an autism spectrum diagnosis in adulthood.

**Methods:**

This retrospective cohort study was extracted from health-administrative data from Quebec (Canada) and included all adults with a first recorded autism spectrum diagnosis between 2010 and 2017 (index date). A Trajectory of psychiatric Diagnoses (TDx) was defined as a succession of categorical states, each corresponding to a medical record of a psychiatric diagnosis. These TDx were analysed from 2002 to 2017, using a state sequence analysis with trimester as time units. For each trimester, we defined the following diagnoses in order of priority: 1) autism spectrum, 2) intellectual disability (ID), 3) schizophrenia, 4) bipolar disorder (BP), 5) depressive disorder (DD), 6) anxiety disorder (AD), 7) attention-deficit/hyperactivity disorder (ADHD), and 8) other psychiatric disorders. The simple Hamming metric was used to measure the dissimilarity between TDx, followed by a hierarchical cluster analysis to categorise similar trajectories.

**Results:**

The study cohort included 2799 adults diagnosed with autism spectrum between 2010 and 2017. Several psychiatric disorders were recorded during the study period, including AD (77.5%), DD (58.0%), schizophrenia (49.4%), BP (48.3%) and ID (33.2%). Results revealed 5 distinct types of TDx. Types 1 and 2, shared by 63.8% and 17.6% of the cohort respectively, represented individuals in younger age groups with similar characteristics, but with very different sequences of psychiatric diagnoses. Slight or sharp increases in diagnoses were observed around 2010, predominantly associated to autism spectrum in Type 1, and to schizophrenia and AD in Type 2. Individuals in Type 4 (6%) were little different from Types 1 and 2, but the TDx showed high prevalence of diagnoses of ID, DD, AD and ADHD, decreasing progressively around the diagnosis of autism. Types 3 and 5 (9.0% and 3.6%), representing middle-aged/older groups, displayed distinctive trajectories of high healthcare use, almost entirely associated with schizophrenia (Type 3), and BD (Type 5).

**Conclusion:**

This study proposes a complementary examination of the multiple pathways to diagnosis experienced by autistic adults, highlighting the need for further investigation into co-occurring psychiatric disorders.

